# PRIMED: PRIMEr Database for Deleting and Tagging All Fission and Budding Yeast Genes Developed Using the Open-Source Genome Retrieval Script (GRS)

**DOI:** 10.1371/journal.pone.0116657

**Published:** 2015-02-02

**Authors:** Michael T. Cummings, Richard I. Joh, Mo Motamedi

**Affiliations:** Massachusetts General Hospital Cancer Center and Department of Medicine, Harvard Medical School, Charlestown, Massachusetts, United States of America; University College London, UNITED KINGDOM

## Abstract

The fission (*Schizosaccharomyces pombe*) and budding (*Saccharomyces cerevisiae*) yeasts have served as excellent models for many seminal discoveries in eukaryotic biology. In these organisms, genes are deleted or tagged easily by transforming cells with PCR-generated DNA inserts, flanked by short (50-100bp) regions of gene homology. These PCR reactions use especially designed long primers, which, in addition to the priming sites, carry homology for gene targeting. Primer design follows a fixed method but is tedious and time-consuming especially when done for a large number of genes. To automate this process, we developed the Python-based Genome Retrieval Script (GRS), an easily customizable open-source script for genome analysis. Using GRS, we created PRIMED, the complete PRIMEr D atabase for deleting and C-terminal tagging genes in the main *S. pombe* and five of the most commonly used *S. cerevisiae* strains. Because of the importance of noncoding RNAs (ncRNAs) in many biological processes, we also included the deletion primer set for these features in each genome. PRIMED are accurate and comprehensive and are provided as downloadable Excel files, removing the need for future primer design, especially for large-scale functional analyses. Furthermore, the open-source GRS can be used broadly to retrieve genome information from custom or other annotated genomes, thus providing a suitable platform for building other genomic tools by the yeast or other research communities.

## Introduction

**Figure 1:**
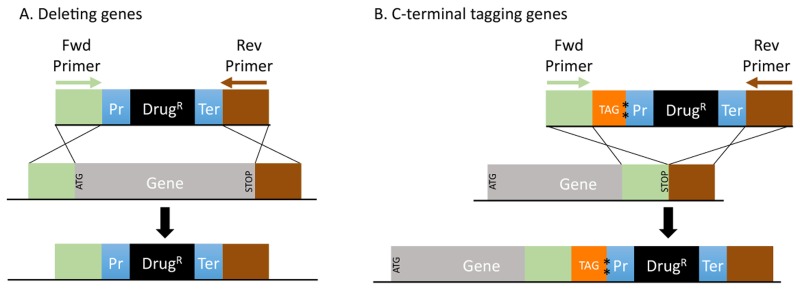
Transformation for deleting or C-terminal tagging genes in yeast. (A) To delete a gene, PCR-generated DNA fragments containing a selectable marker (Drug^R^) flanked by target gene homology (green block, upstream of start codon (ATG), and brown block, downstream of stop codons) are used to transform yeast cells. Recombination between the DNA fragment and the genomic locus deletes the gene and replaces it with the selectable marker. Terminal homologies used in transformation are embedded within especially designed long primers (shown as green and brown arrows) which are used to amplify the transforming DNA fragments from a series of previously described vectors (3,4). Primer design involves selecting the correct regions of homology (length = N basepair) relative to the target gene. For the forward (Fwd) primer, ATG plus N-3 bp upstream of the start codon is selected. For the reverse (Rev) primer, the stop codon and N-3 bp downstream of the gene is selected. For non-coding genes or other features, N bp upstream and downstream of the feature for Fwd and Rev primers is selected, respectively. (B) To add a tag to the C-terminus of a CDS, a Fwd primer (green arrow) containing N bp directly upstream of the stop codon along with Rev primer (depicted in brown as described in A) are used to amplify the fragment shown in this figure by PCR. Homologous recombination integrates the tag (orange) in-frame with the ORF, resulting in a fusion gene. The tag carries its own translation termination codon (shown as two asterisks on top of one another).

The use of budding (*Saccharomyces cerevisiae*) and fission (*Schizosaccharomyces pombe*) yeasts has enabled many groundbreaking discoveries in eukaryotic biology. This is in large part because in these organisms genes can be deleted or tagged easily by transforming cells with PCR-generated DNA fragments, containing a heterologous insert flanked by short (50–100bp) regions of gene homology (Fig. 1) [1, 2]. To generate these DNA fragments, a series of modular vectors carrying different selectable markers and common proteins tags are used as PCR templates [3, 4]. The terminal homologies flanking the DNA inserts are provided by designing long PCR primers, which in addition to carrying the priming sites for these vectors, also carry the homology required for proper integration of these DNA constructs within the target gene. By varying the DNA insert and terminal homologies, perfect gene deletions and in-frame epitope fusions can be constructed easily and quickly (Fig. 1). These techniques also can be coopted to analyze the function of noncoding RNAs (ncRNAs) or non-transcribed regulatory elements in these organisms.

Primer design involves selecting the precise DNA sequence needed for the precise integration of the insert relative to the target gene (Fig. 1). This process requires access to sequence information, often is tedious, slow and error-prone especially if performed for several genes. Currently two websites offer automated primer design for tagging or deleting coding sequences (CDS) for the fission [5] and budding [6] yeasts. These websites are limited in scope in that primers can be obtained for only one gene at a time, deletion primers for noncoding RNAs (ncRNAs) are not provided, and in the case of *S. cerevisiae*, primers for only one lab strain (S288C) can be obtained. Also, these programs do not provide the flexibility to input custom or the most updated version of the yeast genomes. Here we present PRIMED, the complete PRIMEr Database for deleting (CDSs and ncRNAs) and C-terminal tagging (CDSs) the main *S. pombe* and five of the most commonly used *S. cerevisiae* strains.

To generate PRIMED, we developed the Python-based Genome Retrieval Script (GRS), an easily customizable open-source code for retrieving sequence information from annotated yeast or other genomes. PRIMED are accurate, comprehensive and are provided as downloadable Excel files, removing the need for future primer design, especially for large-scale functional analyses. Because of the compact nature of the yeast genomes, we also highlight instances in which deletion of a gene disrupts a part of a neighboring CDS or ncRNA, potentially complicating downstream analyses. Furthermore, the open source GRS can be used to retrieve genome information from other annotated genomes (for example, generating primer databases for other yeast species), thus providing a suitable platform for developing other genomic scripts by the yeast or other research communities. Overall, PRIMED and GRS are useful resources and tools, respectively.

## Materials and Methods

### Implementation

**Figure 2:**
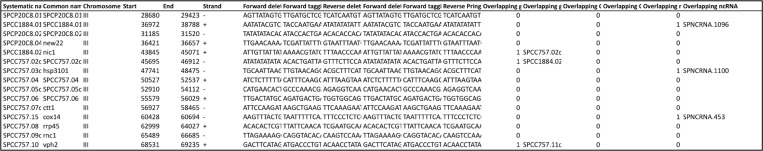
An example of a PRIMED database (fission yeast CDS). In all databases, we provide the systematic and common names, chromosome number, start/end coordinates, transcription strand and forward and reverse primer sequences for each feature under consideration. For deletion databases, also the name(s) and total number of overlapping ORFs, which are disrupted by creating the gene deletion, are indicated.

The databases were generated by a script written in Python 3.3.1. The main advantage of using Python is its large native library. Primers for deleting and C-terminal tagging CDSs and deleting ncRNAs were generated by implementing the following steps:

1. *Calling Input files:* We used the reference genome and annotation files from pombase.org [7] (*S. pombe*), *Saccharomyces* genome database [8] (*S. cerevisiae* S288C) and *Saccharomyces* genome resequencing project [9] (RM11–1A, SK1, W303, and Y55 *S. cerevisiae* strains) as inputs. Inputs are 1) the whole genome sequence file (fa or equivalent format) and 2) the corresponding annotation file (gff3 or equivalent format). All genomic databases used in this study are listed in Table 1. The program begins by calling up its input file as read-only.
2. *Extracting feature information from input files:* GRS first extracts chromosome level information from the sequence file (S1 Appendix and Fig. A in S1 Appendix). Next, it extracts the information of the desired genomic feature (for example CDS, ncRNA, or 3’ UTR) from the annotation file (S1 Appendix and Fig. B in S1 Appendix). For each feature, the program stores the chromosome number, start and end coordinates and transcriptional directionality. Finally, using these coordinates GRS extracts the sequence of the target feature plus N base pairs (bp) of additional sequence, added to its 5’ and 3’ ends. The optimal homology length (N) for HR-mediated integration is different among different yeasts, 80bp and 50bp for *S. pombe* [10] and *S. cerevisiae* [11], respectively. If the gene lies at the end of a chromosome, GRS fills the neighboring sequence, which falls outside of the chromosome, with repetitive N’s In *S. cerevisiae*, we treated pseudogenes and transposable elements as genes.
*Designing forward deletion primer*(Fig. 1A): As the name suggests, this primer is designed for deleting a feature, for example a gene. For CDSs, this primer is the start codon (ATG) plus N-3bp of sequence upstream of the start codon. For ncRNAs or other genomic features, this primer is N bp upstream of the start coordinate. This primer is provided in two versions: 1) the region of homology to the genome only, and 2) the region of homology plus the extra 20bp (CGG ATC CCC GGG TTA ATT AA) sequence for amplification from a pFA6a-based vector [3, 4]. The former can be added to a customized 20+bp sequence for amplification from a user-specific vector construct or PCR amplicon. The latter hybridizes to the multi-cloning site (MCS) on pFA6a- based vectors, which flank the heterologous DNA insert [3, 4]. For deleting 3’ UTRs, a transcriptional terminator must be provided for the gene, otherwise aberrant termination could disrupt gene function. Therefore, a different priming site is used (21 bp, GCG AAT TTC TTA TGA TTT ATG) which amplifies the adh1 terminator found on pFA6a- based vectors [3, 4]. Some recent reports have shown that the 3’ UTRs of gene can play an important role in regulating gene expression [12].
3. *Designing forward C-terminal tagging primer*(Fig. 1B): This primer is used for C-terminal tagging of CDSs and is designed by extracting N bp upstream of the stop codon. We also provide two versions of this primer—with and without the extra 20 bp (CGG ATC CCC GGG TTA ATT AA) sequence for amplification from a pFA6a-series vector [3, 4]
4. *Designing Reverse primer* (Fig. 1A and 1B): This primer is used for both deleting and C-terminal tagging of genes. For CDSs, this primer is the reverse complement of the stop codon plus N-3 bp of sequence downstream of the stop. For ncRNAs or other genomic features, this primer is the reverse complement of N bp downstream of the end coordinate. These primers use GAA TTC GAG CTC GTT TAA AC, the 20bp constant priming site used to amplify DNA from the pFA6a-series vectors.
5. *Creating the number and list of overlapping genes/ncRNAs*: The average distance among genes and ncRNAs is short in yeast [12], thus creating deletions can result in a partial loss of a fragment of a neighboring open reading frame (ORF). In PRIMED, we note instances in which the deletion of a feature impacts the integrity of a neighboring gene, coding sequence (CDS) or ncRNA (S1-S6 Tables, columns M-R).
6. *Primer database*: All primers for each strain are saved as a text file, which we later compiled as an Excel file (Fig. 2). They are presented in two versions plus or minus constant priming sequences found on the pFA6a- based vectors. Each primer output file provides the basic information of the given feature including start/stop coordinates, chromosome identity and direction of transcription. Also, we provide the name of the neighboring CDS or ncRNA which may be disrupted because of the deletion.

**Table 1:** List of yeast strains in the database and their sources for genome and annotation files, as well as their command line inputs.

**Input 1**	**Organism**	**Strain**	**Source**	
0	*S. pombe*	972/ATCC24843	pombase.org [7, 13]	
1	*S. cerevisiae*	S288C	*Saccharomyces* Genome Database [6, 8]	
2	*S. cerevisiae*	RM11–1A	Sanger Institute [9]	
3	*S. cerevisiae*	SK1	Sanger Institute [9]	
4	*S. cerevisiae*	W303	Sanger Institute [9]	
5	*S. cerevisiae*	Y55	Sanger Institute [9]	
**Input 2**	**Feature**		**Input 3**	**Length of Homology**
1	CDS (also generate primers for C-terminal tagging)		50	*S. cerevisiae*
2	ncRNA		80	*S. pombe*
3	3’UTR (only for *pombe*)			
4	tRNA			

In summary, GRS was used to generate long primers for all strains and features listed in Table 1 (S1-S6 Tables).

The Python script GRS is customizable with different input parameters:

>python primer.py INPUT1 INPUT2 INPUT3

INPUT1 is an integer from 0 to 5 which specifies the input genome (0 = *pombe*, 1 = *cerevisiae* S288C, 2 = *cerevisiae* RM11 1A, 3 = *cerevisiae* SK1, 4 = *cerevisiae* W303 and 5 = *cerevisiae* Y55). Other input files can be used with our program with minor modifications. Therefore, GRS gives the user the flexibility to use custom (*e.g.* most updated version of the fission and budding yeast genomes) or other annotated (*e.g. C. albicans*) genomes for analysis.

INPUT2 determines the type of genomic feature to be analyzed by GRS (1 = CDS, 2 = ncRNA, 3 = 3’UTR (only for *pombe*), and 4 = tRNA). GRS scans the provided annotation files (gff3) to extract the genome coordinates and sequence for the specified feature.

INPUT3 determines the desired length of neighboring sequence (N in bp), extracted from the sequence files based on the feature coordinates. This was set to 80 for *pombe* and 50 for *cerevisiae*. This allows the user to change N easily pending application. For example, the primers for *pombe* CDS with 80bp homology (>python primer.py 0 1 80), and *cerevisiae* ncRNA with 50bp homology (>python primer.py 1 2 50) can be generated (S1 Appendix). The resulting output text files are Excel-importable.

The script generates three files for a given analysis (Fig. C in S1 Appendix).

File 1 (the header): In this file, genome coordinates, number of features under analysis and structure of the other output files are provided.

File 2 (the primer database): In this file, primers for deleting or C-terminal tagging each gene/feature are provided in addition to chromosome number, start and end coordinates and direction of transcription for each feature.

File 3 (a check file): In this file, 5’ and 3’ end regions and the entire sequence of the feature are provided for easy verification with the available genome browser tools (S1 Appendix and S1-S6 Tables). We checked our results *via* nucleotide BLAST for accuracy [13].

## Results

### The Databases

PRIMED was constructed for the main *S. pombe* (972 / ATCC 24843) and five of the most commonly used *S. cerevisiae* strains (S288C, RM11–1A, SK1, W303, Y55) (S1-S6 Tables, respectively). For each genome, primers are provided in a separate Excel file. Each Excel file is organized into four sheets, named CDS, ncRNA, 3’UTR (*pombe* only) and tRNA, indicating the genomic feature for which primers were designed. Systemic and common names along with chromosome number, genome coordinates, and Forward and Reverse primers, transcriptional directionality along with potential overlap with neighboring ORFs are provided for each feature. Search for individual features can be performed easily by providing the systemic or common name (for CDSs) in the “Search” feature in Excel. All six databases are provided as downloadable Excel files.

## Conclusions

Here we present PRIMED, the complete PRIMEr Database for deleting and C-terminal tagging the main *S. pombe* and five of the most commonly used *S. cerevisiae* strains. These downloadable Excel files are accurate, comprehensive and also include deletion primers for all ncRNAs. To create PRIMED, we developed the open-source, Python-based Genome Retrieval Script (GRS). GRS uses whole genome and annotation files to extract coordinate and sequence information for any annotated feature. It allows users to extract sequence information from neighboring chromosomal regions at customizable lengths. Slight modifications to GRS can expand its application to custom or other annotated genomes, and enables beginners and advanced users to perform a variety of genomic analyses easily. We believe that GRS can act as a suitable platform for the development of other genomic tools by the scientific community. Overall PRIMED and GRS are valuable sources for the yeast research community, removing any need for future primer design and are a great time-saving resource for large-scale deletion or tagging studies.

## Website

GRS is provided in two downloadable files (S1 File, Genome Retrieval Script (GRS) code, and S2 File, Genome Retrieval Script (GRS) Read Me file) in this paper. PRIMED are provided in S1-S6 Tables. GRS, PRIMED and genome sequence files used to generate PRIMED are available for download at www.massgeneral.org/motamedilab.

## Supporting Information

S1 Appendix Supporting Information text and figures. **Fig. A,** Extracting name and length of a chromosome from genome sequence files. Red bold characters denote extracted information. For *S. pombe*, chromosome number and length is extracted from the sequence file, whereas for *S. cerevisiae*, chromosome length is calculated by counting the number of characters in the chromosome sequence. **Fig. B,** Extracting information about a genomic feature from annotation files. The script scans through the annotation file by “type”, and then extracts chromosome number, start/end coordinates, directionality and the systematic name for each feature. **Fig. C,** A sample of the output files generated using GRS. The output files shown above are for the *S. pombe* CDS database. (A) Header files contain genome coordinates, total number of features under analysis in the genome and structure of other the output files. (B) Primer files show all forward and reverse primers for deleting or tagging genes. The deletion primer databases also show if deleting the gene of interest disrupts neighboring ORFs. (C) The check file shows the 5’ and 3’ end regions of a feature for easy verification. **Fig. D,** Extracting other genomic features using GRS. Upper panel is a screen shot of the Read Me file. The script can generate sequence information for all CDSs, ncRNAs, 3’UTRs and tRNAs and the desired sequence length from neighboring regions. In addition, with minor modification, it can handle other genomic features or can be adopted to analyze another annotated yeast genome. Lower panel is a screen shot of the Python script for GRS. It shows an example of the comments provided with the GRS code. These comments instruct how the code can be modified to analyze custom genomes or other genomic features in an annotated genome.
(DOCX)

S1 File Genome Retrieval Script (GRS) code. (PY)

S2 File Genome Retrieval Script (GRS) Read Me file. (TXT)

S1 Table PRIMED for *S. pombe* (972 / ATCC 24843). (XLSX)

S2 Table PRIMED for *S. cerevisiae* (S288C). (XLSX)

S3 Table PRIMED for *S. cerevisiae* (RM11). (XLSX)

S4 Table PRIMED for *S. cerevisiae* (SK1). (XLSX)

S5 Table PRIMED for *S. cerevisiae* (W303). (XLSX)

S6 Table PRIMED for *S. cerevisiae* (Y55). (XLSX)
