# Supplementary material for: PRIMED: PRIMEr Database for Deleting and Tagging All Fission and Budding Yeast Genes Developed Using the Open-Source Genome Retrieval Script (GRS)
Source: PLoS One. 2015 Feb 2;10(2):e0116657. doi: 10.1371/journal.pone.0116657 (PMC4314071; doi:10.1371/journal.pone.0116657)
Supplement: S1 Appendix — Fig. A, Extracting name and length of a chromosome from genome sequence files. Red bold characters denote extracted information. For S. pombe, chromosome number and length is extracted from the sequence file, whereas for S. cerevisiae, chromosome length is calculated by counting the number of characters in the chromosome sequence. Fig. B, Extracting information about a genomic feature from annotation files. The script scans through the annotation file by “type”, and then extracts chromosome number, start/end coordinates, directionality and the systematic name for each feature. Fig. C, A sample of the output files generated using GRS. The output files shown above are for the S. pombe CDS database. (A) Header files contain genome coordinates, total number of features under analysis in the genome and structure of other the output files. (B) Primer files show all forward and reverse primers for deleting or tagging genes. The deletion primer databases also show if deleting the gene of interest disrupts neighboring ORFs. (C) The check file shows the 5’ and 3’ end regions of a feature for easy verification. Fig. D, Extracting other genomic features using GRS. Upper panel is a screen shot of the Read Me file. The script can generate sequence information for all CDSs, ncRNAs, 3’UTRs and tRNAs and the desired sequence length from neighboring regions. In addition, with minor modification, it can handle other genomic features or can be adopted to analyze another annotated yeast genome. Lower panel is a screen shot of the Python script for GRS. It shows an example of the comments provided with the GRS code. These comments instruct how the code can be modified to analyze custom genomes or other genomic features in an annotated genome. (DOCX) [file pone.0116657.s001.docx]

**Appendix S1**

PRIMED: PRIMEr Database for deleting and tagging all fission and budding yeast genes developed using the open-source Genome Retrieval Script (GRS)

Michael T. Cummings^1^*, Richard I. Joh^1^*, Mo Motamedi^1,2^

1. Massachusetts General Hospital Cancer Center and Department of Medicine, Harvard Medical School, Charlestown, MA, USA

2. Corresponding Author email: [mmotamedi@hms.harvard.edu](mailto:mmotamedi@hms.harvard.edu)

Phone: 617-726-0676

* Equal contribution

**Extracting feature information from input files**

We extract the information of a given genomic feature from the full-genome sequence (.fa or equivalent) and annotation (.gff3 or equivalent) files.

First, GRS extracts the name and length of each chromosome from the sequence file. It creates a list for the header (starting with “>”) and sequence, each of which corresponds to a chromosome. For the *S.* *pombe* genome, chromosome number and length is extracted by separating different delimiters as shown in Figure A. In this instance, 4^th^ and 6^th^ elements are chromosome number and length, respectively. For the *S.* *cerevisiae* genomes, chromosome number is in the 5^th^ bracket as shown in Figure A. Chromosome length is determined by counting the number of characters for each chromosome sequence file. Note that each sequence file can have a different header line, so the script may need minor modifications to fit the user’s need.


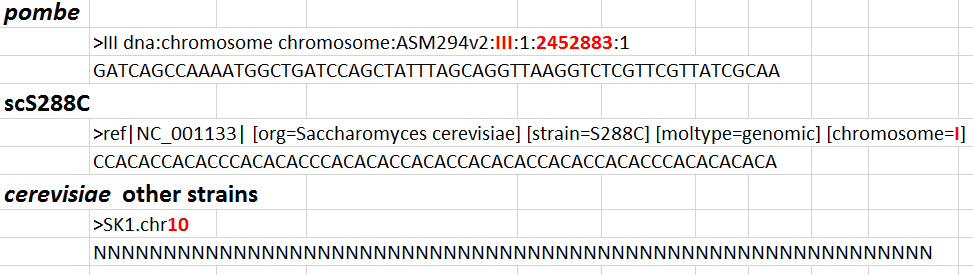


**Figure A**. Extracting name and length of a chromosome from genome sequence files. Red bold characters denote extracted information. For S. pombe, chromosome number and length is extracted from the sequence file, whereas for S. cerevisiae, chromosome length is calculated by counting the number of characters in the chromosome sequence.

Then the script reads the annotation file to look for a specific genomic feature. Typically, the annotation file has 9-columns as shown in Figure B. Among them, the program extracts chromosome number, type of feature, start/end coordinates, strand and attributes (shown as bold red colors in Figure B). The name of chromosomes, types of feature, and attributes are often annotation-specific, and should be optimized if custom annotation files are used. The current script can be used to extract information about genes (whole transcripts), coding sequences, ncRNAs, 3’ untranslated regions (UTRs), 5’ UTRs and tRNAs. In *cerevisiae* genome files, in addition to coding sequences (CDSs) ‘genes’ also include transposable elements and pseudogenes. 5’ UTRs are only annotated in the genome file of scS288C strain.


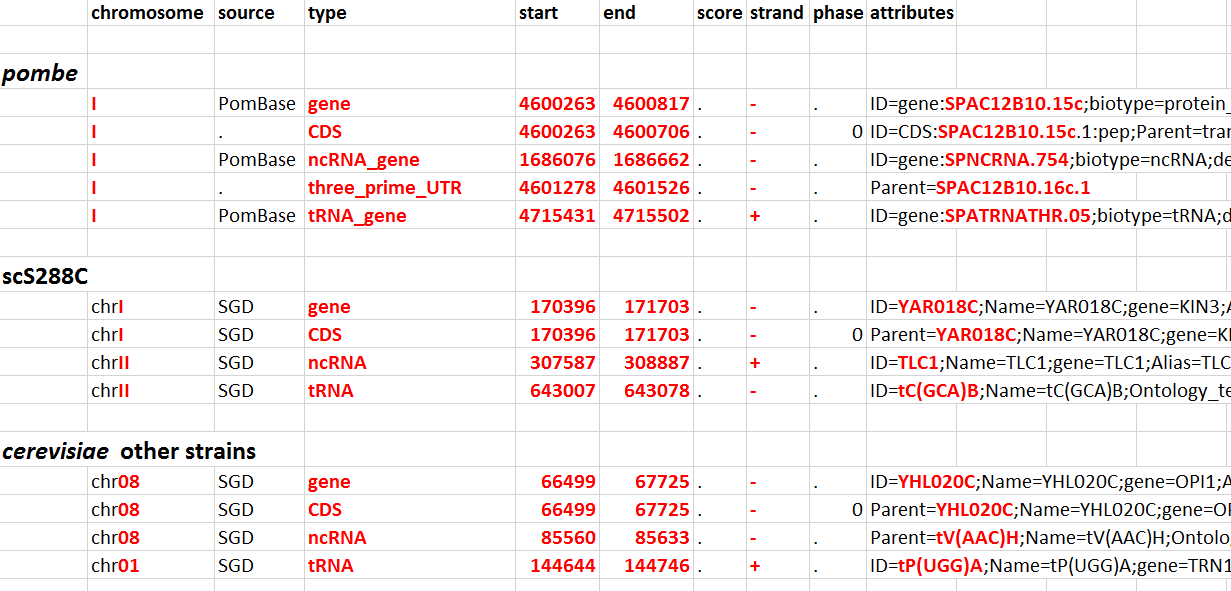


**Figure B**. Extracting information about a genomic feature from annotation files. The script scans through the annotation file by “type”, and then extracts chromosome number, start/end coordinates, directionality and the systematic name for each feature.

**File Structure**

The script creates 3 output files for each genomic feature.

1. Stat files: These files store the basic information of a feature in the genome. Data in these files show chromosome name and length, and the number of genes/CDSs/ncRNAs/features. These files also show the file structure of the other two output files (see below).
2. Primer files: This is the file containing all the primers as tab-delimited text files. Each column represents the following:

Systematic name

Common name or alias

Chromosome number

Start coordinate

End coordinate

Strand

Forward deletion primer

C-terminus tagging forward primer (only for CDS)

Reverse deletion primer

Forward deletion primer for pFA6a-based Vectors [1,2]

C-terminus tagging forward primer for pFA6a-based Vectors (only for CDS)

Reverse deletion primer for pFA6a-based Vectors

Number of overlapping ORFs

List of overlapping ORFs

Number of overlapping CDSs

List of overlapping CDSs

Number of overlapping ncRNAs

List of overlapping ncRNAs.

1. Check files: These files stores other information of genomic features such as

Systematic name

Common name or alias

Chromosome number

Start coordinate

End coordinate

Strand

First 3bp

Last 3bp

N bp upstream and N bp in the 5’ end

N bp in the 3’ end and N bp downstream

Feature sequence

where N is the length of overhang.


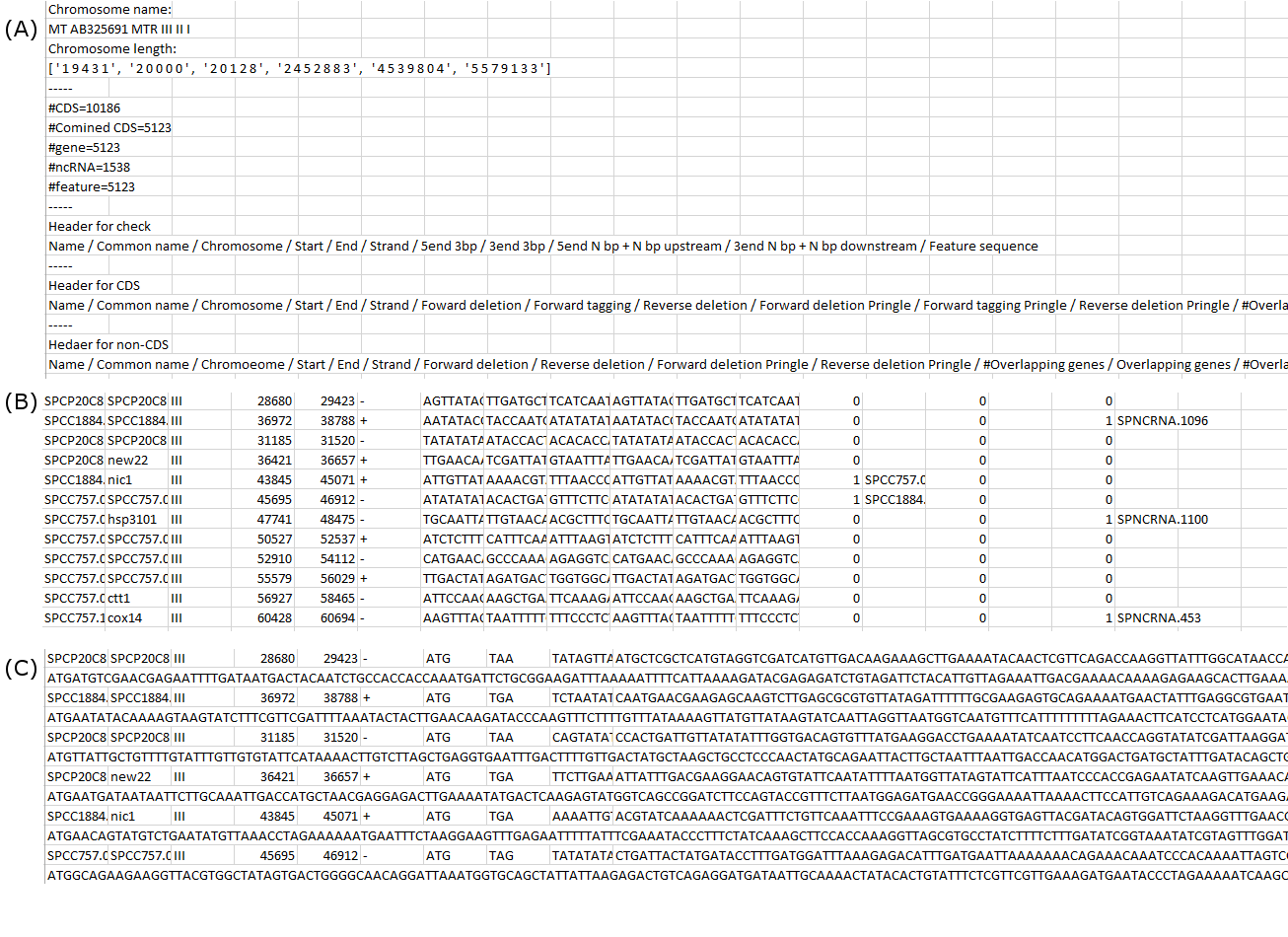


**Figure C**. A sample of the output files generated using GRS. The output files shown above are for the S. pombe CDS database. (A) Header files contain genome coordinates, total number of features under analysis in the genome and structure of the other output files. (B) The Primer files show all forward and reverse primers for deleting or tagging genes. The deletion primer databases also show if deleting the gene of interest disrupts neighboring ORFs. (C) The check file shows the 5’ and 3’ end regions of a feature for easy verification.

**Commands for the database**

As described earlier, the script can generate custom sequences which can fit the user’s need. The command lines used to generate all databases presented in this paper are as follows:

*pombe* CDS: >python primer.py 0 1 80

*pombe* ncRNA: >python primer.py 0 2 80

*pombe* 3’UTR: >python primer.py 0 3 80

*pombe* tRNA: >python primer.py 0 4 80

scS288C CDS: >python primer.py 1 1 50

scS288C ncRNA: >python primer.py 1 2 50

scS288C tRNA: >python primer.py 1 4 50

scRM11 1A CDS: >python primer.py 1 1 50

scRM11 1A ncRNA: >python primer.py 1 2 50

scRM11 1A tRNA: >python primer.py 1 4 50

scSK1 CDS: >python primer.py 1 1 50

scSK1 ncRNA: >python primer.py 1 2 50

scSK1 tRNA: >python primer.py 1 4 50

scW303 CDS: >python primer.py 1 1 50

scW303 ncRNA: >python primer.py 1 2 50

scW303 tRNA: >python primer.py 1 4 50

scY55 CDS: >python primer.py 1 1 50

scY55 ncRNA: >python primer.py 1 2 50

scY55 tRNA: >python primer.py 1 4 50.

**Extracting other genomic features**

Figure D shows how GRS can be used to extract information about other genomic features. By uncommenting two lines, GRS can extract information from repetitive DNA elements, rRNA and 5’UTR in *S. pombe*. The sequence and genome coordinates along with a desired length of neighboring sequence can be extracted with slight modifications to GRS.


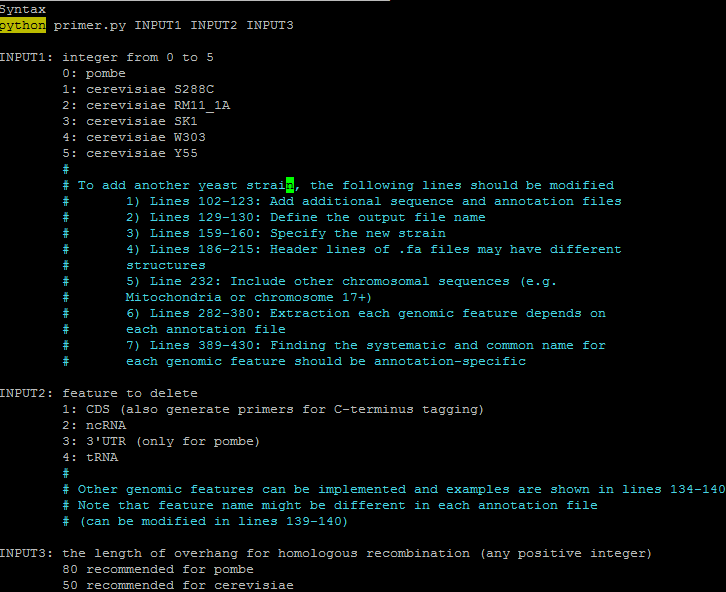

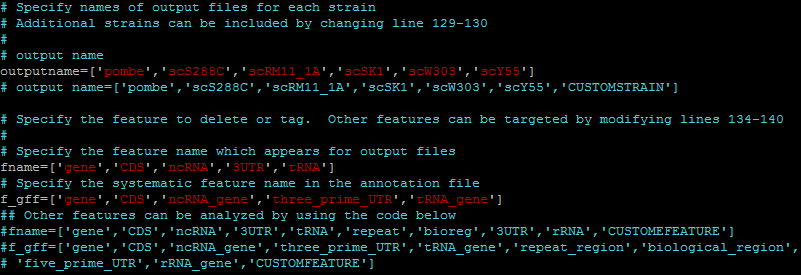


**Figure D**. Extracting other genomic features using GRS. Upper panel is a screen shot of the Read Me file. The script can generate sequence information for all CDSs, ncRNAs, 3’UTRs and tRNAs and the desired sequence length from neighboring regions. In addition, with minor modification, it can handle other genomic features or can be adopted to analyze another annotated yeast genome. Lower panel is a screen shot of the Python script for GRS. It shows an example of the comments provided with the GRS code. These comments instruct how the code can be modified to analyze custom genomes or other genomic features in an annotated genome..

**References**

1. Longtine MS, McKenzie A, Demarini DJ, Shah NG, Wach A, et al. (1998) Additional modules for versatile and economical PCR-based gene deletion and modification in *Saccharomyces cerevisiae*. Yeast 14: 953–961.

2. Bähler J, Wu JQ, Longtine MS, Shah NG, McKenzie A, et al. (1998) Heterologous modules for efficient and versatile PCR-based gene targeting in *Schizosaccharomyces pombe*. Yeast 14: 943–951. A
